# Predictors of religious participation of older Europeans in good and poor health

**DOI:** 10.1007/s10433-016-0367-2

**Published:** 2016-03-12

**Authors:** Agnieszka Sowa, Stanisława Golinowska, Dorly Deeg, Andrea Principi, Georgia Casanova, Katherine Schulmann, Stephania Ilinca, Ricardo Rodrigues, Amilcar Moreira, Henrike Gelenkamp

**Affiliations:** 1 Center for Social and Economic Research (CASE), Al. Jana Pawła II 61/212, Warsaw, Poland; 2 Institute of Labour and Social Studies, Bellottiego 3B, Warsaw, Poland; 3 Collegium Medicum Jagiellonian University, ul. Grzegórzecka 20, Cracow, Poland; 4 VU University Medical Centre, De Boelelaan 1089aHV, Amsterdam, The Netherlands; 5 National Institute of Health and Science on Ageing (INRCA), via S. Margherita 5, 60124 Ancona, Italy; 6 European Centre for Social Welfare Policy and Research, Berggasse 17, 1090 Vienna, Austria; 7 Institute of Social Science, University of Lisbon, Av. Professor Aníbal de Bettencourt 9, 1600-189 Lisbon, Portugal

**Keywords:** Older people, Ageing, Health status, Morbidity, Religious participation

## Abstract

Religious attendance is an important element of activity for older Europeans, especially in more traditional countries. The aim of the analysis is to explore whether it could be an element contributing to active ageing as well as to assess differences between the religious activity of older individuals with and without multimorbidity defined as an occurrence of two or more illnesses. The analysis is conducted based on the SHARE database (2010–2011) covering 57,391 individuals 50+ from 16 European countries. Logistic regressions are calculated to assess predictors of religious activity. Results point that religious activity often occurs in multimorbidity what could be driven by the need for comfort and compensation from religion. It is also significantly correlated with other types of social activities: volunteering or learning, even among the population with multimorbidity. There is a positive relation between religious activity and age, although its effect is weaker in the case of multimorbidity, as well as being female. Mobility limitations are found to decrease religious participation in both morbidity groups and might be related to discontinuation of religious practices in older age. The economic situation of older individuals is an insignificant factor for religious attendance. Religious attendance can be an element of active ageing, but also a compensation and adaptation to disadvantages occurring in older age and multimorbidity. At the same time, religious activities are often provided at the community level and targeted to population in poorer health.

## Introduction

Strategic documents of the European Union (EU) and the World Health Organization (WHO) indicate the activity of the older population is one of the main factors of active ageing underlining its impact on decreasing the economic costs of ageing, including the need for extensive healthcare, postponing disability, and reducing costly long-term care (WHO 2002). Typically, activities that might contribute to more active ageing include higher employment opportunities, involvement in life-long learning, physical and cultural activities (WHO 2002). In the European societies that are more traditional, such as Poland or Italy, participation in the labour market, life-long learning, or even volunteering at an older age is low, with cultural and social activities closely linked to religious participation, which remains a dominant activity for older people. Various studies note religious involvement as an important element of healthy ageing, related to quality of life and compensating for social isolation, poor family networks, and incapacities in various fields of life (Koenig et al. 1988; Benjamins 2004; Woźniak 2012).

The analysis presented in this study examines the hypothesis of religious activity as an element of successful ageing, notifying differences in motivations for religious involvement depending on health status.

## Background

Studies of religious attendance and health underline the importance of religious participation not only due to the intrinsic, internalised, and spiritual value of religious beliefs and participation, but also due to the extrinsic values of religious life related to networking, social support, and the cultural life of the religious community (Jarvis and Northcott 1987; Sloan et al. 1999; Huguelet and Koenig 2009, Koenig et al. 2014; Krause and Hayward 2014).

### Health and religious participation

The discussion on the relation between religious participation and health has been held for many years without a strong conclusion on the direction of the relationship (Hummer et al. 2004).

There is evidence of the impact of religious involvement, especially religious activities of a public character, on adult mortality risks (Levin 1994; Hummer et al. 2004; Huguelet and Koenig 2009). Some studies find a positive impact of religion on mental and physical health (Levin 1994, 2012; Siegel 2012) indicating that a greater level of religiosity is positively related to better health outcomes: lower morbidity and better psychological well-being (Levin 1994). It is argued that an active involvement in religious activities might even improve longevity (Hummer et al. 1999; Hybels et al. 2012; Siegel 2012). A study of the poor older population in Connecticut (Jarvis and Northcott 1987) concludes that religiousness and attendance was positively correlated with a reduction in mortality. Other studies find that different dimensions of religious involvement have a protective effect against a functional decline among the older population (Park et al. 2008; Hybels et al. 2012). The study by Park et al. (2008) concludes that attending religious services is related to lower levels of functional limitations and decreases the risks of developing limitations in the instrumental activities of daily living. A similar relation has not been found for private religious practices, such as watching and listening to religious media and prayer. The authors also state that the mechanism by which religious involvement appears to influence mortality includes aspects of social integration, social regulation, and psychological resources. Huguelet and Koenig (2009) indicate that religious practices might prevent patients from developing symptoms of depression and, if the symptoms do occur, recovery is quicker.

Important explanations of the positive, bilateral relation between religious participation and health refer to the life-style factors, social behaviours, psychological factors, and social support that are given in a religious community (Jarvis and Northcott 1987; Levin 1994; Iannaccone 1998; Siegel 2012; Krause and Hayward 2014). Particular attention is given to health behaviours, self-perception, and social support.

Religious beliefs promote the adoption of a healthy life-style, governing strict rules on the use of alcohol, tobacco, drugs, diet, and sexual behaviours. In general, values of different religions discourage risk-taking behaviours, which are important risk factors for morbidity and mortality (i.e. alcohol abuse and smoking). Benjamins and Brown (2004) argue that religion might be related not only to the avoidance of risky health behaviours, but also positively related to health awareness and preventive care use. Their study shows that controlling for possible confounders of age and sex, physical and mental health, and socio-economic status, religious individuals are more likely to receive flu vaccinations, cholesterol screenings, and prostate screenings (males).

The psychological effects of belief systems, rituals, and faith stimulating the locus of control and self-perception are also important for health status (Levin 1994). Beliefs in particular religions might encourage a peaceful state of mind or a greater sense of optimism due to a feeling of sense of purpose in life. The psychological effects of participation in rituals might have a great impact on emotions, creating an effect that might be referred to as a ‘placebo’ effect. Adversely, in some cases, the belief system might produce guilt or low self-esteem (Huguelet and Koenig 2009).

An important factor for a positive bidirectional relation between religion and health is social networking and social support. Religious participation is related to lesser feelings of isolation, greater social participation, and closer family ties. For single people, involvement in religious institutions may protect against loneliness later in life by integrating older adults into larger and supportive social networks (Woźniak 2012; Rote et al. 2013). Studies by Krause (2002), Koenig et al. (2014), and Krause and Hayward (2014) point that religious participation is related to gratefulness and more social support that is positively related to better self-concept, optimism, and better health in older age.

Finally, religion operates as a cushion. It mitigates the impact of stressful events, such as illness, work problems, involuntary residential changes, or hospitalisation. Huguelet and Koenig (2009), analysing the situation of older patients with neurologic symptoms, note that religion has been the most important coping factor, being a source of comfort, helping patients reframe poor conditions or loss into a positive situation, and providing a feeling of purpose or meaning.

### Demographic, social, and economic correlates of religious participation

The character of religious participation has changed over the past decades, with an observable decrease in church participation accompanied by a turn to less institutionalised forms of sharing values and norms (Luckmann 2011). At the same time, participation in religious communities varies depending on traditions, religious human capital, socialisation within the family, social networks, community relations, and relations with peers, as well as by social and demographic characteristics (Cornwall 1989; Ammerman and Roof 1995).

### Life course trends in participation

A life course pattern of involvement in religious activities can be identified. Bahr (1970) described four life course patterns of religious involvement: traditional pattern of the highest involvement in childhood and older age; stable involvement throughout life and lack of relation between ageing and religious participation; highest religious involvement related to family life and religious education of children; decrease in religious practices in older age that accompanies drawing back from social activities. Stable involvement in religious activities throughout life supports the continuity theory of ageing pointing to internal and external coherence of individual behaviours in older age and consistency of behaviour throughout life (Atchley 1989). Many recent studies point to a “u” shape pattern of involvement in religious activities with the highest levels of participation in the early years of life and for older people, though participation for the oldest old (80 or more) tends to decrease due to mobility limitations in favour of private religious practices (prayer) (Wink and Dillon 2001; Heineck 2001; Timonen et al. 2011).Involvement in religious activities in older age might be an element of adaptation to losses in health and social networks and selection of a meaningful activity that gives a sense of purpose in older age, providing a compensation in situation of losses (Baltes and Baltes 1990; Freund and Baltes 1998). The higher religious participation of older people might also be related to compensation of perceived lower social security (Borowik 2002; Woźniak 2012), existential fears, and adaptation to insecurity while approaching the ends of their lives. Participation in religious practices and communities might also be a substitute for the vanishing social networks of older people (Woźniak 2012). On the other hand, despite that a similar age pattern of religious involvement is observed in all countries in Europe and the US (Smith 2009), a cohort effect should be accounted for pointing that cohorts reaching older ages tend to be religious throughout their lives (Woźniak 2012).

### Gender differences

Level of religious participation is typically higher among women. This pattern has been observed for different age groups (Iannaccone 1998; Heineck 2001; Timonen et al. 2011). Among the explanations of higher female attendance in religious activities might be their involvement in the religious socialisation of children and better opportunities for time allocation to religious activities due to a lower involvement in the labour market, especially in traditional societies (Levin 1994; Heineck 2001). However, a higher labour market participation of women in contemporary societies might result in a lower involvement in religious life than in the past (Ammerman and Roof 1995). At an older age, higher religiosity is observed for the widowed (Heineck 2001). Additionally, higher attendance in religious services, accompanied by declarations of receiving comfort and strength from religion, is observed more often among women than men (Timonen et al. 2011).

Marital status and family life might also play a role in religious attendance. Ammerman and Roof (1995) show that single men are more likely to be involved in non-religious activities, while single women tend to be more often involved in religious activities. Married couples are also more inclined to participate in organised religious activities than their single counterparts. Attendance is also higher in traditional families than in non-traditional families, such as single or stepparent parent families (Petts 2015).

### Education and income

The results of research on religious participation and level of education are complex. In some studies, religiousness and attendance are found to stimulate better educational achievement, work activity, better labour market performance, higher income (Lipford and Tollison 2003), and lower involvement in deviant activities (i.e. crime, alcohol, and drugs) (Iannaccone 1998; Heineck 2001; Keister 2011). Iannaccone (1998) notes that the character of religious involvement is different depending on education level, with more orthodox religious values more often observed among the less educated and the poor. Other empirical analyses support a secularisation hypothesis that higher education decreases individual religious attendance, noting a strong negative relation between attendance and higher education across religious groups (Halicka and Halicki 2002; Pędich 2002; Woźniak 2012; Zhang 2012). Higher education and higher incomes are also constraints to religious attendance due to high opportunity costs, as time devoted to religious practices could be used for labour-related purposes (Ammerman and Roof 1995; Heineck 2001; Woźniak 2012). In the future in less traditional societies, the negative correlation between higher levels of education and lower religious participation might also appear in the later life due to reaching older age by less religious cohorts (Hungerman 2011; Woźniak 2012).

Participation in religious activities might be important for the active and healthy ageing being related to positive emotions, better self-perception, social networking, and support; however, involvement in activities might be dependent upon a variety of factors. Previous research shows that the sense of engagement, a purpose in life, generosity, and involvement are prominent predictors of healthy ageing, even more important than a health status itself (Reichstadt et al. 2007). The sense of purpose and social networking might be related both to religious involvement (Keonig et al. 2014; Krause and Hayward 2014) and to health status. Religious involvement in older age in different morbidity status might be an element of compensation of losses in health, social networks, lower security, and adaptation to changing life circumstances in multimorbidity related to age. On the other hand, for religious individuals morbidity might not be an obstacle in continuation of their religious involvement in older age giving a meaning to life that arises from internal continuity. To test these relations, the article investigates patterns of religious involvement of people with and without morbidity to identify resources (functional capacity, age, education, social involvement, other) that stimulate the engagement in religious activities. The definition of multimorbidity refers to the disablement process as described by Verbrugge and Jette (1994) in which multimorbidity is an expression of the chronic conditions and impairments experienced by older people, while religious involvement is an intra-individual factor that might prevent further disablement.

## Methods

The analysis uses data from the Survey of Health, Ageing and Retirement in Europe (SHARE) of 2010 and 2011 (Wave 4, Release 1.1.1), a cross-national survey study covering individuals aged 50+ from 16 European countries. The analysis of religious activities covers 57,391 individuals. Individuals who have not answered the question on religious attendance or answered “don’t know” were excluded from the original SHARE sample (300 respondents, 0.52 % of the total SHARE sample). More than every fifth person in the sample suffers from multimorbidity and every tenth individual reports mobility limitations (Table 1). Dementia occurs occasionally. Females constitute over half of the sample and more frequently suffer from a higher number of morbidities. Almost half of the sample is below the age of 65, while every fifth person is over the age of 75. In this age group, multimorbidity is the most frequent. Additionally, in the 50–64 age group, almost every third person suffers from two or more morbidities. More than half of individuals live in households consisting of two members. Approximately 80 % of the sample has a primary or secondary education, while 20 % reports higher educational attainment. More than half of the sample is retirees and 27 % is either employed or self-employed. Unemployment or receiving some type of sickness benefit is much less common. When social activities are in question, every fifth respondent is involved in sports and club activities or is providing some type of care. Voluntary work and educational activities are less common, with 16 % of individuals reporting involvement in some type of voluntary activity. 13 % of the sample reports participating in religious activities and less report participating in educational activities.

**Table 1:** Sample characteristics: total sample and by morbidity status

Variables	Categories	Total sample *N* = 57391		Less than 2 morbidities *N* = 44122		2 morbidities or more *N* = 13269		*p* value*
		*N*	%	*N*	%	*N*	%	
Mobility limitations (1+)		6663	*11.62*	3021	*6.85*	3642	*27.46*	*0.000*
Poor mental health		23933	*42.64*	15,820	*36.57*	8113	*63.06*	*0.000*
Dementia		700	*1.22*	367	*0.83*	333	*2.51*	*0.000*
Demographic								
Gender								
Male		24904	*43.39*	19,442	*44.06*	5462	*41.16*	*0.000*
Female		32487	*56.61*	24,680	*55.94*	7807	*58.84*	
Age								
50–64		27241	*48.48*	23,308	*54.21*	3933	*29.81*	*0.000*
65–74		16631	*29.60*	12,346	*28.71*	4285	*32.47*	
75+		12318	*21.92*	7341	*17.07*	4977	*37.72*	
Marital status								
Single		3164	*5.59*	2517	*5.79*	647	*4.92*	*0.000*
Married		40390	*71.38*	32,020	*73.70*	8370	*63.70*	
Divorced		4948	*8.74*	3776	*8.69*	1172	*8.92*	
Widowed		8082	*14.28*	5132	*11.81*	2950	*22.45*	
Household size								
Single		11839	*20.63*	8164	*18.50*	3675	*27.70*	*0.000*
2 persons		31895	*55.57*	24,601	*55.76*	7294	*54.97*	
3 persons+		13657	*23.80*	11,357	*25.74*	2300	*17.33*	
Human capital								
Education								
Primary		21314	*39.04*	15,233	*36.17*	6081	*48.75*	*0.000*
Secondary		21794	*39.92*	17,291	*41.05*	4503	*36.10*	
Higher		11486	*21.04*	9596	*22.78*	1890	*15.15*	
Socio-economic								
Labour market								
Retired		32331	*56.35*	22,840	*51.78*	9491	*71.55*	*0.000*
Employed or self-employed		15726	*27.41*	14,348	*32.53*	1378	*10.39*	
Unemployed		1923	*3.35*	1621	*3.68*	302	*2.28*	
Sick or disabled		2091	*3.64*	1223	*2.77*	868	*6.54*	
Other		5301	*9.24*	4076	*9.24*	1225	*9.24*	
Income quartile**								
1st		14499	*25.26*	10,437	*23.65*	4062	*30.61*	*0.000*
2nd		14689	*25.59*	10,736	*24.33*	3953	*29.79*	
3rd		14680	*25.58*	11,557	*26.19*	3123	*23.54*	
4th		13523	*23.56*	11,392	*25.82*	2131	*16.06*	
Social participation								
Learning activities		6736	*11.74*	5879	*13.32*	857	*6.46*	*0.000*
Informal care provision		13539	*27.76*	10,563	*27.58*	2976	*28.41*	*0.093*
Sports, clubs		14718	*25.65*	12,309	*27.90*	2409	*18.16*	*0.000*
Voluntary work		9132	*15.91*	7533	*17.07*	1599	*12.05*	*0.000*
Religious activity		7449	*12.98*	5705	*12.93*	1744	*13.14*	*0.031*

*Source* own calculations based on SHARE data 2010–2011

Italic values indicate that Chi-square test is significant at the 0.05 level

* *p* values for Chi-square tests of association between the listed variables (i.e. demographic, human capital, and other characteristics vs. morbidity)

** Income quartiles are calculated separately for each country, adjusting for differences in income distribution in each country

### Participation in religious activities

Participation in religious activities is assessed by the question if an individual *has attended*/*taken part in activities of a religious organisation* (*church, synagogue, mosque*) *in the past twelve months*. This question does not specify the types of activities individuals might be involved in. While it might consider various types of activities (including community meetings and voluntary activities), it is assumed that attendance in public religious services is the primary activity. The dependent variable is binary, identifying if an individual has or has not attended religious activities.

### Multimorbidity assessment

The analysis is performed for two groups based on morbidity level. Multimorbidity is assessed using an indicator based on the number of self-reported morbidities. The list of morbidities includes myocardial infarction, stroke, or cerebrovascular disease; diabetes or high blood sugar; chronic pulmonary diseases, including pneumonia, emphysema, or asthma; arthritis, including osteitis and rheumatic disease; cancer, including leukaemia and lymphoma (without minor skin cancers); gastric or duodenal ulcer; Parkinson’s disease; cataracts; and hip, femoral, and other types of fractures. Multimorbidity is assessed as reporting two or more of the illnesses specified in the survey (van den Akker et al. 1997; Marengoni et al. 2011). Multimorbidity was selected as the best operationalisation of health status for the purpose of behavioural analysis of health (van den Akker et al. 1997).

### Predictors of religious activity

Potential predictors of religious activity include health status, demography, human capital, labour market position, income, and social participation. Models controlling for functional health status and excluding these types of predictors are presented and compared. This is to control for the fact that health status might not only be on the pathway between morbidity and disability (Verbrugge and Jette 1994), but also might be an important determinant of religious participation, especially in the case of poor functional abilities (Sloan et al. 1999).

*Functional abilities and mental health* is assessed by the ability to perform basic activities of daily living, mental health, and the occurrence of dementia. To assess mobility limitations, a binary variable of reporting at least one limitation in activities of daily living has been created. The mobility items specified in the survey include walking 100 m; sitting for 2 h; getting up from a chair after sitting for a long period; climbing several flights of stairs without resting; climbing one flight of stairs without resting; stooping, kneeling, or crouching; reaching or extending arms above shoulder level; pulling or pushing large objects, such as a living room chair; lifting or carrying weights over 10 pounds (5 kg), such as a heavy bag of groceries; and picking up a small coin from the table (Jagger et al. 2011). Mental health is assessed using the Euro-D scale (Prince et al. 1999), assigning poor mental health if the number of symptoms is greater than three. A separate binomial is created for occurrence of cognitive dysfunctions, such as dementia, e.g. Alzheimer’s disease. The presence of cognitive illnesses was assessed in the survey through self-reporting.

Basic *demographic factors* include age, sex, marital status, and household size. Three age groups have been differentiated: 50–64, 65–74, and 75+ . Households have been categorised into three groups depending on size: single, two members, and three or more members. Marital status has been categorised into single, married, divorced, and widowed.

*Human capital* is measured by level of education. An original SHARE variable corresponding to the ISCED-97 scale was simplified into the three categories of primary, secondary, and tertiary education.

*Socio*-*economic status* is measured by labour market position and income level, with the latter calculated separately for each country.

*Social participation* is assessed by a set of dichotomous variables on participation in volunteering, educational activities, clubs and sports, as well as the provision of regular but informal care of any type (to a spouse, children, or others) in the previous year.

The main part of the analysis is multivariate country-pooled logistic regression models identifying the predictors of religious participation by morbidity level, which is a grouping variable. Coefficients indicating the strength of the relation in the logistic model should not be simply compared between the models (Allison 1999); thus, the average marginal effects of each model are presented and discussed. Models have been calculated separately for individuals with and without multimorbidity to present differences in the set of predictors of religious activity depending on morbidity status. Following, a control variable of mobility limitations has been introduced, and again models have been compared to assess whether mobility might be a significant factor explaining religious activity depending on morbidity and if it impacts other relations. Presenting models separately for each morbidity status allows for simple and clear understanding of the possible set of relations for individuals with or without multimorbidity.

## Results

The participation in religious activities among the older population in the case of multimorbidity is slightly higher than the participation of those without multimorbidity (Table 1). Participation is more frequent among females with multimorbidity when compared to healthy females, individuals before the retirement age with multimorbidity when compared to ‘younger’ older people without morbidities, the better educated in poor health when compared to those healthier, and the employed or self-employed and wealthier individuals with multimorbidity when compared to individuals without illnesses (Table 2).

**Table 2:** Participation in religious activities in older age: total sample and by morbidity status

Variables	Categories	Total sample	Less than 2 morbidities		2 Morbidities or more	
		%	%	*p* value*	%	*p* value*
Mobility limitations (1+)		11.15	11.06	*0.001*	11.23	*0.000*
Poor mental health		13.21	13.26	*0.157*	13.10	*0.303*
Dementia		8.86	7.63	*0.002*	10.21	*0.109*
Demographic						
Gender						
Male		10.49	10.57	*0.000*	10.20	*0.000*
Female		14.89	14.79		15.20	
Age						
50–64		11.65	11.53	*0.000*	12.36	*0.028*
65–74		14.43	14.49		14.26	
75+		14.20	15.11		12.86	
Marital status						
Single		11.50	11.28	*0.000*	12.36	*0.029*
Married		12.97	12.92		13.18	
Divorced		9.16	8.74		10.49	
Widowed		15.38	16.23		13.90	
Household size						
Single		13.65	13.46	*0.000*	14.07	*0.000*
2 persons		11.96	12.02		11.76	
3 persons+		14.78	14.52		16.04	
Human capital						
Education						
Primary		13.19	13.19	*0.000*	13.21	*0.000*
Secondary		11.80	11.84		11.61	
Higher		14.71	14.39		16.35	
Socio-economic						
Labour market						
Retired		13.38	13.65	*0.000*	12.73	*0.000*
Employed or self-employed		10.73	10.53		12.84	
Unemployed		10.14	10.06		10.60	
Sick or disabled		10.81	10.79		10.83	
Other		19.09	19.14		18.49	
Income quartile**						
1st		13.21	13.29	*0.001*	13.00	*0.975*
2nd		13.59	13.71		13.26	
3rd		12.96	12.88		13.29	
4th		12.09	11.92		13.00	
Social participation						
Informal care provision		14.92	14.88	*0.000*	15.05	*0.000*
Sport, clubs		16.00	15.74	*0.000*	17.31	*0.000*
Voluntary work		27.24	27.07	*0.000*	28.08	*0.000*
Educational activity		17.64	17.09	*0.000*	21.35	*0.000*
Country						
Poland		43.22	42.74	*0.000*	44.78	*0.000*
Austria		19.85	19.70		20.39	
Netherlands		18.13	18.31		16.81	
Switzerland		16.60	16.49		17.27	
Slovenia		15.25	14.71		17.61	
Spain		14.66	14.21		16.00	
Portugal		14.37	12.08		21.04	
Hungary		13.78	13.40		14.51	
Sweden		13.41	13.29		14.01	
Belgium		12.84	13.15		11.84	
Germany		12.13	12.47		10.88	
Italy		11.63	11.63		11.65	
Denmark		9.99	10.01		9.88	
Czech Republic		7.49	6.89		9.33	
France		7.39	7.13		8.27	
Estonia		4.89	4.67		5.32	

*Source* own calculations based on SHARE data 2010–2011

Italic values indicate that Chi-square test is significant at the 0.05 level

* *p* values for Chi-square tests of association between the dependent variable (religious participation) and other characteristics and morbidity

** Income quartiles are calculated separately for each country, adjusting for differences in income distribution in each country

Among the main predictors of religious participation of older people are sex, age, functional ability, and active participation in social life (Table 3).

**Table 3:** Comparison of predictors of older people religious activities depending on morbidity and not controlling for functional limitations, average marginal effects

Variable	No multimorbidity (<2 diseases)	Multimorbidity (≥2 diseases)
	dx/dy (SE)	dx/dy (SE)
Poor mental health (ref. good m.h.)	−0.000 (0.003)	−0.002 (0.006)
Dementia (ref. no dementia)	−0.049*** (0.013)	−0.049** (0.016)
Female (ref. male)	0.036*** (0.003)	0.051*** (0.006)
Age (ref. 50–64)		
65–74	0.026*** (0.005)	0.019* (0.009)
75+	0.052*** (0.007)	0.028** (0.010)
Marital status (ref. single)		
Married	0.007 (0.007)	0.020 (0.014)
Divorced	−0.028*** (0.008)	−0.026 (0.016)
Widowed	0.011 (0.010)	−0.009 (0.016)
Household size (ref. single)		
2 persons	−0.010 (0.008)	−0.038** (0.015)
3 persons+	0.013 (0.008)	−0.008 (0.013)
Education (ref. primary)		
Secondary	−0.011** (0.004)	−0.018** (0.007)
Higher	0.006 (0.005)	0.011 (0.010)
Labour market position (ref. retired)		
Employed or self-employed	−0.005 (0.005)	0.006 (0.012)
Unemployed	−0.001 (0.009)	−0.012 (0.019)
Sick or disabled	−0.002 (0.010)	−0.001 (0.014)
Other	0.021*** (0.006)	0.019 (0.012)
Income (ref. 1st quartile)		
2nd quartile	0.001 (0.005)	0.009 (0.009)
3rd quartile	−0.006 (0.005)	0.012 (0.010)
4th quartile	−0.016*** (0.005)	−0.006 (0.010)
Informal caregiving (ref. no caregiving)	0.014*** (0.004)	0.013 (0.007)
Sports, clubs (ref. no clubs activity)	0.024*** (0.004)	0.021* (0.009)
Volunteering (ref. no volunteering)	0.164*** (0.006)	0.169*** (0.014)
Educational activity (ref. no religious act.)	0.029*** (0.005)	0.031* (0.013)

The model controls for country differentials

*Source* own calculations based on SHARE data 2010–2011

* *p* < 0.05, ** *p* < 0.01, *** *p* < 0.001

Females are more likely to participate in religious activities than males and the result is significant in both morbidity groups. The likelihood of religious involvement increases with age for both morbidity groups. The significance of effect is smaller in case of no multimorbidities. In both groups of older people, with and without multimorbidity, dementia negatively affected religious participation, while the role of mental health was not significant.

In the group with no multimorbidity, religious practices are negatively correlated with divorce. In the group with multimorbidity, this relation is not significant.

Living in a two-person household decreases the probability of involvement in religious activities, but only among individuals with multimorbidity. In the group with no multimorbidity, household composition is an insignificant determinant of religious participation.

Despite level of morbidity, secondary education decreases the likelihood of religious practices when compared to primary education. This relation is not observed for individuals with a higher educational attainment. The labour market status of older individuals is, in most cases, an insignificant predictor of religious involvement, despite morbidity level. The only exception is the category of ‘other’, which includes the labour market inactive that are often involved in family care or household work. The probability of religious involvement of this group is higher than that of the retired, but only in the case of healthier individuals. Similarly, income level is insignificant for participation in religious practices at an older age, with the only exception decrease in the probability of religious participation for those with the highest incomes and no multimorbidity.

Social participation is a significant predictor of religious participation. Involvement in sports, clubs, educational activity, and, especially, volunteering are significantly and positively related to participation in religious activities despite morbidity level. In the case of the provision of informal care, the positive relation is less significant for the multimorbidity group than for healthier people; however, it is still an important predictor of religious participation.

Including mobility items (Table 4) shows that mobility limitations are an important limitation to religious participation, decreasing probability of religious attendance, while other relations depicted earlier are similar. This is observed in people with and without multimorbidity.

**Table 4:** Comparison of predictors of older people religious activities depending on morbidity and controlling for functional limitations, average marginal effects

Variable	No multimorbidity (<2 diseases)	Multimorbidity (≥2 diseases)
	dx/dy (SE)	dx/dy (SE)
Mobility limitations (1+) (ref. no limitations)	−0.017*** (0.003)	−0.016*** (0.003)
Poor mental health (ref. good m.h.)	0.001 (0.003)	0.003 (0.006)
Dementia (ref. no dementia)	−0.038* (0.016)	−0.040* (0.018)
Female (ref. male)	0.036*** (0.003)	0.050*** (0.006)
Age (ref. 50–64)		
65–74	0.027*** (0.005)	0.021* (0.009)
75+	0.055*** (0.007)	0.034** (0.010)
Marital status (ref. single)		
Married	0.008 (0.007)	0.019 (0.014)
Divorced	−0.028*** (0.008)	−0.026 (0.016)
Widowed	0.012 (0.010)	−0.007 (0.016)
Household size (ref. single)		
2 persons	−0.009 (0.008)	−0.032* (0.015)
3 persons+	0.014 (0.008)	−0.001 (0.014)
Education (ref. primary)		
Secondary	−0.011** (0.004)	−0.018* (0.008)
Higher	0.006 (0.005)	0.010 (0.010)
Labour market position (ref. retired)		
Employed or self-employed	−0.005 (0.005)	0.006 (0.012)
Unemployed	−0.001 (0.009)	−0.013 (0.018)
Sick or disabled	0.003 (0.011)	0.007 (0.015)
Other	0.021*** (0.006)	0.019 (0.011)
Income (ref. 1st quartile)		
2nd quartile	0.001 (0.005)	0.008 (0.009)
3rd quartile	−0.006 (0.005)	0.011 (0.009)
4th quartile	−0.016*** (0.005)	−0.007 (0.010)
Informal care giving (ref. no caregiving)	0.014*** (0.004)	0.015* (0.007)
Sports, clubs (ref. no clubs activity)	0.023*** (0.004)	0.018* (0.008)
Volunteering (ref. no volunteering)	0.163*** (0.003)	0.166*** (0.014)
Educational activity (ref. no religious act.)	0.029*** (0.005)	0.031* (0.013)

The model controls for country differentials

Source: own calculations based on SHARE data 2010–201

* *p* < 0.05, ** *p* < 0.01, *** *p* < 0.001

## Discussion

Analysing religious participation based on multimorbidity is not easy given the complexity of the relation between religious participation and health (Sloan et al. 1999) as well as the multidimensionality of religiosity (Jarvis and Northcott 1987; Sloan et al. 1999) and the complexity of religious participation. On the one hand, participation could be driven by deep faith, but also by other needs, including those driven by age and feelings of frailty related to poor health or the need for cultural or social participation when the church might be one of the few locally available institutions fulfilling the need (Kędziora 2013).

The analysis of the predictors of religious participation of older people confirms the previous findings of typically higher activity among women (Iannaccone 1998; Heineck 2001; Timonen et al. 2011) with a possible explanation including a greater involvement in religion throughout life related to the socialisation of children or grandchildren and better opportunities to allocate time for religious activities due to a decreased involvement in the labour market, especially in more traditional religious societies (Levin 1994; Heineck 2001). This effect could also be attributable to the adaptation and compensation effect as women not only have a higher attendance in religious services, but also declare finding comfort and strength from religion more often than men (Timonen et al. 2011). The observed higher religiosity of widowed women of an older age is often explained by the effect of finding comfort and consolation from religious practices and beliefs in times of loss. This study, however, does not support this hypothesis.

The research confirms the importance of age for the involvement in religious practices (Heineck 2001; Smith 2009; Timonen et al. 2011; Kędziora 2013). The increased participation of the oldest cohorts might be related to various factors, including compensating for decreased involvement in family life or social networks and more available time, as well as the cohort effect of older individuals being more traditional and oriented towards religion. Lower significance of age among people with multimorbidity might imply lower capabilities of involvement in religious activities due to health limitations and point to the possible discontinuation of public religious practices in favour of continuity of private practices for religious individuals (Sloan et al. 1999).

The analysis indicates differences in the likelihood of religious participation depending on marital status and multimorbidity. The lower probability of participation among healthier divorced people can be explained by their exclusion from the religious community in most religious denominations. This effect is not visible in case of poorer health when adaptation and compensation motivations might be of greater importance then the rules of religious exclusion. Other types of marital status were insignificant despite the results of other research pointing to stronger support and social networks for married couples in religious communities (Wilcox and Wolfinger 2008; Petts 2015). At the same time, people living in two persons household, most likely couples, in case of morbidity are found to be less motivated to participate in religious activities than single couples. This might imply that in case of poor health they find support at home, from their spouse and are less likely to search for psychological support outside, in church.

The relations between educational attainment and religious participation partly confirm the findings of Hungerman (2011), where higher educational attainment was negatively related to religious participation later in life. Here, against primary education, the relation is significant only for secondary education. For higher educated no motivation is found for religious activity in case of higher morbidity.

The poor involvement in religious activities of the working population has been found in other studies (Heineck 2001) with an explanation of higher opportunity costs for such involvement. Religious participation is also in conflict with employment, especially for professional groups and those with higher incomes due to the scarcity of time available for religious practices. This concept has been only partially confirmed in this study, where higher incomes are found to decrease the likelihood of religious participation for the group of older people without multimorbidity. Only the labour market inactive that are involved in home activities are correlated with religious participation. This could occur for individuals living in families that are more traditional or communities that have more opportunities to allocate time for religious activities.

The analysis confirms that participation in social activities is positively related to religious activity. Previous studies show a positive relation between volunteering and religious attendance (Smith 1994) though the effect differentiates between denominations—an element not tackled by current analysis. In addition, members of the religious community are more willing to be involved in the voluntary activities supported by their churches (Wilson and Janoski 1995). Involvement in clubs or educational activities might also be stimulated by religious groups.

Finally, the results point to the health constraints of religious attendance. Sloan et al. (1999) underline that functional disabilities might be a significant constraint to public religious participation and the results confirm this relation. This study also adds dementia and other cognitive disorders to the list of constraints of religious participation.

This study adds to the existing literature by comparing behaviours in the two morbidity groups. While the level of religious participation for those with morbidities is only slightly higher than that for those with good health, there are subtle differences in predictors between the morbidity groups. They point to lower obstacles in religious participation among individuals with higher morbidity, with age being a less important predictor of participation and family situation and family decomposition (divorce) being insignificant. These results might be related to the greater need for comfort and consolation of individuals with poor health who face higher insecurity due to their poor health. Such results might support the selectivity, optimisation, and compensation theory pointing to religious involvement being one of possible adaptation mechanisms in less secure situation of poor health. On the other hand, even in the case of multimorbidity, religious attendance is positively correlated with other types of social participation, which might indicate that the health deterioration is not an obstacle to religious practices among more active individuals or that religious organisations often are the providers of cultural or educational activities to people in poor health, which enables their participation.

SHARE is a unique database providing evidence on, among other types of activity, the religious participation of the older population and allowing for comparison with information on health status, morbidity, and functional abilities, as well as information on the social and economic status of individuals. However, the definition of religious activity in the SHARE questionnaire is blurred, with unknown types of religious activities and a reference to a broad timeframe of the year preceding the survey. As a result, it might not only cover regular attendance in religious practices, but might include more occasional religious attendance or participation in activities organised by churches, but not related to religious practices, such as volunteering and participation in church clubs or educational meetings. Another drawback of the survey question is that it does not differentiate between denominations, as the level of involvement in public religious activities might differ between denominations, i.e. being higher for Catholics and Muslims and lower for Anglo-Saxon Protestants (Heineck 2001). This is a field for further study. The imprecise definition of religious participation might result in slightly different participation statistics across countries than in other surveys. For example, compared to other European research (Smith 2009; Eurobarometer 2010), the SHARE statistics indicate a higher frequency of religious participation in the Netherlands and a lower frequency of participation in Italy and Portugal. An analysis of religious involvement of older people across countries would be an interesting field for further research. Acknowledging differences in the level of participation depending on morbidity, there is also a space for studying relations with severity of diseases.

## Conclusion

The analysis shows that along other activities, older Europeans participate in religious activities, which could be related to a need for comfort or by cultural or social needs. The religious participation could be driven by spiritual and psychological reasons, but also by the cultural and institutional offers of religious organisations. The occurrence of multimorbidity differentiates religious participation. This should be interpreted bearing in mind that religious activities are often oriented towards those sick and in pain and account for mobility issues, including individuals unable to leave their home or care facility. The wide range of activities of churches and religious organisations is specifically aimed at older and ill people, and religious services are typically provided to older persons at the community level, which increases their accessibility and might be combined with educational or cultural activities.
